# Agronomic performance of cocoa production type systems in Colombia

**DOI:** 10.1371/journal.pone.0337624

**Published:** 2025-12-03

**Authors:** Héctor Eduardo Hernández-Núñez, Juan Carlos Suárez, Hernán J. Andrade, Angie Paola Bernal Núñez, David Ricardo Gutiérrez, Gustavo Adolfo Gutiérrez, Isabel Gutiérrez-Montes, V. Ernesto Méndez, Fernando Casanoves

**Affiliations:** 1 Programa de Doctorado en Ciencias Naturales y Desarrollo Sustentable, Facultad de Ciencias Agropecuarias, Universidad de la Amazonia, Florencia, Caquetá, Colombia; 2 Centro de Investigaciones Amazónicas CIMAZ Macagual Cesar Augusto Estrada González, Grupo de Investigaciones Agroecosistemas y Conservación en Bosques Amazónicos- GAIA, Colombia; 3 Programa de Doctorado en Ciencias Agrarias, Facultad de Ingeniería Agronómica, Universidad del Tolima, Ibagué, Tolima, Colombia; 4 Programa de Ingeniería Agroecológica, Facultad de Ingeniería, Universidad de la Amazonia, Florencia, Caquetá, Colombia; 5 CATIE – Centro Agronómico Tropical de Investigación y Enseñanza, Turrialba, Costa Rica; 6 Institute for Agroecology (IFA), University of Vermont, Burlington, Vermont, United States of America; University of the West Indies, TRINIDAD AND TOBAGO

## Abstract

Socio-economic and livelihood conditions influence the design of cocoa-based agroforestry systems (AFS), as they determine the selection of accompanying species and the overall productivity of the system. These arrangements contribute to income diversification, enhanced food security and increased resilience to climate-related impacts. However, achieving these benefits depends to a large extent on the capabilities of rural households, which are shaped by their human, social, cultural, political, financial, natural, and infrastructural assets. This study analyzes the incidence of rural household type, according to livelihood strategies on the agronomic performance and co-product generation of cocoa crops established in agroforestry systems, in the departments of Caquetá, Huila and Meta in Colombia. Capital endowments for cocoa production, as well as the intensity and frequency of shade canopy species associated with cocoa, were determined in 83 rural households. Then, 112 sampling plots of 1000 m^2^ were established, where 20 variables of information on floristic composition, forest structure of the AFS, state of pest attack and diseases, and cocoa bean production were taken. It was found that the agroforestry characteristics of the cocoa crop and its production differed between rural household types and departments. A total of 176 species associated with cocoa were found, but only 89 were recognized by the farmers. These species were grouped into 17 uses, the most frequent being self-consumption, shade, wood, and sale (for human food). The livelihood strategy and assets for cocoa production had a high impact on the agronomic performance of the crop. Households with more experience, technology, knowledge and dedication to the crop obtained higher cocoa bean yields. These results affirm that the management and efficiency of cocoa-based AFS depend on the articulation of social processes and the design of the AFS.

## Introduction

Cocoa (*Theobroma cacao* L.) is a high-value crop for tropical countries [1]. This crop supports around five million smallholder farming families worldwide [2] and covers approximately 11 million hectares in the humid tropics [3]. Despite its environmental and economic potential, cocoa production faces several challenges, including low yields, pest and disease pressure, and limited access to technology and markets. In Colombia, cocoa has high environmental, social, and economic relevance [4,5]. It sustains the livelihoods of approximately 65,000 smallholder families [6], who manage cocoa plots between 1 and 3 ha [7]. Colombian cocoa beans have been recognized by the International Cocoa Organization as fine and aromatic [8]. Moreover, cocoa plays a strategic role in rural development as an alternative to illicit coca cultivation [7]. This importance is reflected in a 104.8% increase in production in the last 15 years, from 30,357 t in 2006–62,158 t in 2022 [9], of which approximately 95% is exported [10].

Globally, cocoa is mostly cultivated under agroforestry systems (AFS), which, in addition to cocoa beans, provide multiple economic and environmental benefits [11]. These systems contribute to biodiversity conservation [12,13], generate food and timber products that enhance food security and household income [11,14–21], improve soil properties [16,22], and reduce erosion and nutrient leaching [11]. Although a trade-off may occur between cocoa yield and shade tree density, this balance is often compensated by the productivity of associated crops [23]. In Colombia, about 90% of cocoa is produced under AFS [4], and these systems play a key role in strengthening the livelihood strategies and overall well-being of cocoa-dependent families [17,24].

Although shade tree species provide multiple benefits for smallholder livelihoods, they can also generate certain disadvantages that should be further analyzed to improve the efficiency and sustainability of cocoa-based agroforestry systems [25]. From an agronomic perspective, several studies indicate that higher levels of shade and tree species diversity may increase pest and disease incidence, leading to reduced cocoa yields [26–29]. Likewise, Van Der Wolf et al. [25] report that some tree species compete strongly for water and nutrients or generate excessive shade, both of which can negatively affect productivity. Beyond agronomic aspects, social factors also influence the sustainable management of cocoa in AFS. Melo [30] highlights that, despite the existence of local ecological knowledge among farmers, the potential uses and management options of shade canopy species are often underutilized, which limits the benefits that these species can provide. Consequently, producers may lose opportunities to achieve high-yielding systems [31] and to maximize the ecosystem services derived from these species [23].

Traditional cocoa management practices in Colombia are characterized by low levels of technological application [32], which contribute to higher pest and disease incidence [33] and can lead to yield reductions of up to 70% [34,35]. Similar challenges have been widely reported in smallholder cocoa systems across tropical regions, where limited access to innovation, training, and technical assistance constrains productivity and sustainability [2,17,36]. Consequently, Colombia’s contribution to global cocoa production decreased from 1.4% to 1.1% between 1970 and 2020 [37], a trend influenced both by local productivity constraints and by the expansion of cocoa cultivation in other producing countries. According to the National Department of Statistics [38], 78.27% of Colombian cocoa producers operate at a low technological level, 27.87% at a medium level, and less than 1% at a high level. These low levels of technology adoption limit improvements in productivity and household income derived from cocoa bean product [39].

This situation jeopardizes the sustainability of AFS and, therefore, affects the well-being of producer families [2]. The optimal development of cocoa crops requires the convergence of different factors. For example, Hernández-Núñez et al. [21] found that the synchrony of factors such as knowledge of the crop, time dedicated to its management, careful selection of other species grown with cocoa and planting density affects the agronomic condition, determining cocoa production and the generation of other products. Also, Salazar-Díaz and Tixier [40] show that the negative effects of competition, which can lead to lower productivity in some species, are compensated by complementarity and facilitation among species, allowing higher productivity at the system level. Finally, Hernández-Núñez et al. [21] indicates that a high endowment of human, cultural and physical capital allows rural households to improve the agronomic conditions of cocoa cultivation.

Despite the recognition of cocoa as a strategic crop in agroforestry systems in Colombia, there is a significant gap in the understanding of how the socio-productive characteristics of rural households, especially their livelihood strategies and capital endowment, affect the agronomic conditions of the crop and the use of co-products associated with shade species. This knowledge gap limits the development of differentiated interventions that promote not only productivity but also sustainability in cocoa-based agroforestry systems. This study adopts a socioecological approach, aiming to identify the key factors that influence cocoa performance in AFS by integrating agronomic variables, local knowledge, and the community capitals framework. By analyzing how different types of rural households configure and manage their production systems, the study contributes to evidence-based public policy and technical support aligned with local realities.

Consequently, the objective of this study was to analyze how different types of rural households, defined according to their livelihood strategies, influence the agronomic performance and co-product generation of cocoa crops established in AFS in Colombia. Our hypothesis posits that households with a greater endowment of capitals (human, natural, financial, social, political, cultural, and built) would exhibit higher agronomic performance and a more diversified use of co-products derived from shade species. In this research, we addressed the following questions: 1) What is the agronomic performance of cocoa cultivation across different rural household types? 2) Which shade canopy species are most commonly associated with cocoa cultivation? 3) How do rural household types perceive and use the co-products of shade species within cocoa AFS? and 4) What is the relationship between the capital endowment of cocoa-producing households and the agronomic performance of their cocoa crops? This approach represents an innovation by linking social dimensions and community capital analysis with agronomic indicators, providing evidence to strengthen sustainable cocoa-based agroforestry systems and to guide livelihood-oriented rural development strategies in tropical regions.

## Materials and methods

### Area and population of study

The study was conducted in the departments of Caquetá, Huila and Meta, which represent three natural regions of Colombia. These departments were selected based on their contrasting dynamics of cocoa production and socio-economic contexts. In Caquetá and Meta, cocoa production has experienced significant fluctuations due to pest and disease outbreaks, reduced institutional support, and the expansion and subsequent eradication of illicit coca crops, which influenced farmers’ livelihood strategies and encouraged the recovery of old plantations and the establishment of new ones [41,42]. This process intensified after 2013 with the peace process, favoring the expansion of cocoa as a sustainable alternative for rural households. In contrast, Huila has presented a more consolidated and technically supported cocoa sector, with production variations associated with market dynamics and the presence of fine-flavor cocoa varieties with high quality potential [43,44]. In each department, municipalities with significant participation in cocoa cultivation were prioritized to capture the diversity of production systems and household conditions within these regions.

In the three departments, climatic conditions show significant differences. In Caquetá, warm and humid climates predominate, with temperatures around 25 °C and annual rainfall exceeding 3,000 mm; in Huila, climates range from temperate to warm sub-humid, with temperatures between 12 °C and 28 °C and rainfall from 1,000–2,000 mm; in Meta, the municipalities exhibit warm and humid climates with temperatures between 22 °C and 24 °C and rainfall up to 3,000 mm [45].

The present research addressed a mixed approach [46]. In each sampled unit (a rural household dedicated to cocoa cultivation), an interview (quantitative information) and a survey were conducted; in addition, a sampling plot was established in the cocoa field to analyze the agroforestry structure of the crop, the incidence and severity of pests, and cocoa yield (quantitative information). Eighty-three rural households, corresponding to Cocoa Farmers (n = 18), Livestock-Cocoa-Off-Farm Income (n = 24), Coffee-Cocoa Farmers (n = 4), Diversified (n = 24) and External Cocoa Farmers (n = 13) typologies were selected from the departments of Caquetá, Huila and Meta (Table 1). The condition to be included was to have a cocoa production system with at least 3 years of plantation. This selection was based on the work of Hernández-Núñez et al. [47], who investigated the role of cocoa in the livelihoods of 406 rural households in the departments of Caquetá, Huila, Meta and Santander in Colombia. Household classification was based on the livelihood strategy, which was determined according to their productive activities. This was accomplished through the following variables: number of productive activities, ratio of the area of each productive activity in relation to the productive area of the farm, and ratio of income from each productive activity with the total income of the farm were used.

**Table 1:** Types of rural households according to livelihood strategies in the departments of Caquetá, Huila and Meta, Colombia.

Household typology	Characteristics of the typology
Cocoa Farmers: CocF	Cocoa cultivation is the most important livelihood for these households, 90% depend economically on the production of this crop and the area planted in cocoa (3.6 ha) represents 91% of the productive area of the households. This type of household develops other agricultural activities such as banana production.
Livestock-Cocoa-Off-Farm Income: GaCocExF	This group is characterized by the fact that 91% of their income is derived from livestock for milk production and fattening of steers (39%), cocoa (33%), and off-farm income (21%). Seventy-eight percent and 19% of the household’s productive area (24 ha) is devoted to pastures for livestock and cocoa plantations, respectively. Other productive activities of these households include poultry production.
Coffee-Cocoa Farmers: CofCocF	Households in this group have configured their livelihood strategies around coffee and cocoa crops. The average area planted with coffee (2.3 ha) and cocoa (2.1 ha) represents 40 and 43% of the total productive area of the household, respectively. The economic income derived from coffee and cocoa cultivation corresponds to 51 and 30% of total household income, respectively.
Diversified Farmers: DF	Forty-nine percent of their income is generated by productive activities other than coffee, livestock or cocoa; the latter represents 31% of household income. The main livelihoods include growing bananas, avocados, oranges, tangerines and the production of fish, pigs and poultry. Agricultural and livestock livelihoods contribute approximately 29 and 20% of household income, respectively.
External Cocoa Farmers: ExCocF	Households in this group are characterized by 66% of their income coming from external activities and 31% from cocoa growing. The area planted in cocoa (2.65 ha) represents 94% of the total productive area of households. In 20% of these households, cocoa cultivation is still in the growing stage, so there is no cocoa bean production to date.

Source: [47]

For each producer surveyed, informed consent was obtained and formally signed. Also, all the participants were over 18 years old. The present research was approved by the Ethics Committee of Universidad de la Amazonia, Colombia. This approval guarantees that the research complies with fundamental ethical principles, including respect for the rights of participants, and confidentiality of information. In addition, it supports the scientific integrity of the study, ensuring that its procedure complies to national and international standards in academic research, contributing to the credibility and validity of the results obtained. The data collection was carried out between 03/11/2020 and 14/12/2021.

### Agroforestry and cocoa production conditions in rural household types

In the 83 rural households, 112 sampling plots of 1000 m^2^ (50 × 20 m) were established, where 20 variables on agroforestry and production were registered (7 variables on density of shade canopy species to cocoa trees, 6 on diversity, 6 on state of pest and disease attack on the crop and cocoa bean yield) (Table 2). Two sample plots were established in certain households, as they had two cocoa lots, which were spatially separated and had different age and design characteristics.

**Table 2:** Variables of floristic composition, crop structure, pest and disease attack status and cocoa bean production to study cocoa crops in Caquetá, Huila, and Meta, Colombia.

Component	Variable	Units	References
Density of shade canopy species to cocoa trees	CT: Number of cocoa trees	Individuals ha^-1^	[21,48–50]
	CS: Accompanying Individuals		
	TS: Individuals Timber		
	MS: Individuals Musaceae		
	EFS: Individuals of Food Species		
	FSE: Individuals Fabaceae		
	OS: Other Accompanying Individuals		
Pest and disease attack and damage	IMi: Incidence of *Moniliophthora roreri,*	%	[29,51,52]
	IMa: Incidence of *Monalonion dissimulatum*		
	PSMd: Percentage of severity of *Monalonion dissimulatum*		
	PSEMr: Percentage of external severity of *Moniliophthora roreri*		
	PSIMr: Percentage of internal severity of *Moniliophthora roreri*		
	ESEPh: Percentage of external severity of *Phytophthora spp*		
Diversity	RCS: Richness of accompanying individuals		[48–50]
	SWSCS: Shannon Weaver index of accompanying individuals		
	RUCS: Richness of potential uses		
	SWUCS: Shannon Weaver index of potential uses		
	Cocoa tree companion species density	%	
	Frequency of species accompanying the cocoa tree		
Production	Yield	kg ha^-1^ year^-1^	[21,53]

### Asset endowment for cocoa production system in the types of rural households

Cocoa production system conditions were addressed using the community capital framework approach [54,55]. This approach allows communities and community development efforts to be analyzed through the lens of seven asset-based characteristics that collectively define a community’s potential for positive change [56]. In the present study, the community capital approach was used at the production level (cocoa farming), analyzing specific cocoa production assets. The following variables used in the work of Hernández-Núñez et al. [47] were analyzed: Cultural Capital: a. experience in cocoa cultivation (years), b. dedication to cocoa farming activities (hours/week), c. identity with cocoa cultivation, d. motivations when planting cocoa; Natural Capital: e. cocoa area (ha); Human Capital: f. cocoa trainings (number), g. cocoa knowledge (1−5); Built Capital: h. technological level for cocoa cultivation (low, Medium, high); Social and Political Capital: i. participation in cocoa associations (yes/no); Financial Capital: j. Cocoa dry bean production (kg household year^-1^), and k. annual cocoa income (dollars) (supplement).

### Intensity and frequency of recognized companion species

The survey applied to each rural household asked about the species they had in the cocoa crops and the uses they gave them. From the list of species mentioned by the rural households, the intensity of use and frequency of mention was quantified; this was done following the formulas used by Zavala-Sanchez et al. [57], as follows:

Equation 1


$$\text{Intensity}\;\text{of}\;\text{use}\;=\;\frac{\text{Number}\;\text{of}\;\text{uses}\;\text{of}\;\text{species}\;n\;\text{by}\;\text{all}\;\text{rural}\;\text{households}}{\text{Total}\;\text{number}\;\text{of}\;\text{uses}\;\text{of}\;\text{all}\;\text{species}\;\text{and}\;\text{all}\;\text{rural}\;\text{households}}\times \text{100}$$

Equation 2


$$\text{Frequency}\;\text{of}\;\text{mention}\;=\;\frac{\text{Number}\;\text{of}\;\text{mentions}\;\text{of}\;\text{species}\;n\;\text{from}\;\text{all}\;\text{uses}\;\text{and}\;\text{all}\;\text{informants}}{\text{Total}\;\text{number}\;\text{of}\;\text{mentions}\;\text{of}\;\text{all}\;\text{species}\;\text{for}\;\text{all}\;\text{uses}\;\text{and}\;\text{from}\;\text{all}\;\text{informants}}\times \text{100}$$

### Data analysis

Field data were tabulated in an Excel template (supplementary information). Subsequently, all statistical analyses were performed in the statistical software InfoStat [58] and R Studio [59]. An analysis of variance was conducted to compare the density of shade canopy species to cocoa trees, diversity, state of pest and disease attack on the crop and cocoa bean yield (agroforestry and production variables) among rural household types at the department level. Continuous variables were analyzed by linear mixed models [60], using the typology by department as the fixed effect and the municipality as the random effect, for pest and disease variables, as well as cocoa bean yield, the cocoa clone was included as an additional random effect to account for genetic variability. Discrete variables were analyzed by generalized linear mixed models using Poisson distribution [61]. Ordinal variables were analyzed with contingency tables [62]. Comparisons of means were performed with Fisher’s LSD test (p < 0.05). A contingency table analysis was carried out to determine the behavior of the intensity of use and frequency of mention of the shade canopy species of the cocoa crop among rural households at the department level.

The relationship between community capital endowment and cocoa production, including the density of shade tree species associated with cocoa trees, species diversity, the incidence of pest and disease attacks, and cocoa bean yield, was analyzed using Spearman’s correlation analysis [62]. Finally, a co-inference analysis was conducted to examine the correlations between the set of capital endowment variables and the set of cocoa production variables.

## Results

### Agronomic conditions of the crop and asset endowment for cocoa production in rural household types

We found that households of the same type present different agroforestry and production characteristics of cocoa cultivation among departments, reflecting diverse productive and sociocultural conditions (Table 3). Significant differences (p = 0.0020) in cocoa bean yield were found between household types and departments. CocF and GaCocExF households in the department of Meta had the highest cocoa bean yields, with 1225 and 997 kg ha^-1^ yr^-1^, respectively. The GaCocExF households and the ExCocF households in Caquetá presented the lowest yields with 705 and 645 kg ha^-1^ yr^-1^, respectively.

**Table 3:** Agronomic variables of cocoa crops in rural household types in the departments of Caquetá, Huila and Meta (Colombia).

Variable	Units	Caquetá				Huila					Meta				P-value
		CocF	DF	ExCocF	GaCocExF	CocF	CofCocF	DF	ExCocF	GaCocExF	CocF	DF	ExCocF	GaCocExF	
**Cocoa Bean Yield**															
Yield	kg ha^-1^ year^-1^	675.3 ± 197.74bc	686.74 ± 84.32bc	644.6 ± 139.83c	705.1 ± 61.03bc	806.18 ± 88.43bc	865.58 ± 161.46abc	816.62 ± 93.22bc	844.03 ± 105.7bc	805.3 ± 139.83bc	1223.63 ± 84.32a	829.27 ± 93.22bc	797.15 ± 114.17bc	996.6 ± 93.22ab	0.0020
*Density of species categorized by use*															
CT	Number	735 ± 148.13ab	683.46 ± 54.06b	662.6 ± 84.53b	738.57 ± 45.93ab	1022.92 ± 83.96a	905 ± 128.77ab	877.78 ± 83.28ab	1082.14 ± 116.26a	925 ± 131.59ab	946.36 ± 81.17a	770.44 ± 73.17ab	676.17 ± 78.73b	792.33 ± 75.23ab	0.0030
CS		205 ± 82.69ab	289.23 ± 45.66ab	67.4 ± 17.46d	259.52 ± 32.25ab	248.75 ± 40.91ab	415.25 ± 117.99a	211.11 ± 40.13ab	182.86 ± 39.46b	151.75 ± 43.4bc	79.73 ± 13.87 cd	190.22 ± 36.19b	285 ± 66.23ab	186.11 ± 35.42b	<0.0001
TS		82.5 ± 61.13abc	154.62 ± 44.82a	39.6 ± 18.67bcd	99.29 ± 22.68ab	45.42 ± 13.8bc	18.75 ± 10.01cde	38.89 ± 13.67 cd	73.57 ± 29.16abc	40 ± 21.08bcd	10 ± 3.28e	21.22 ± 7.53cde	46.33 ± 19.91bc	11.78 ± 4.25de	<0.0001
MS		22.5 ± 25.15abc	41.54 ± 18.14ab	4 ± 2.94c	68.81 ± 23.6ab	147.08 ± 66.62a	320 ± 250.86a	96.67 ± 50.59a	76.43 ± 45.38ab	71.25 ± 55.98ab	18.36 ± 8.77bc	73.78 ± 38.64ab	118.33 ± 75.82a	66.56 ± 34.87ab	0.0037
EFS		30 ± 28.27	51.15 ± 18.83	10.8 ± 6.54	41.19 ± 11.95	40.83 ± 15.67	48 ± 31.87	68.89 ± 30.43	22.86 ± 11.54	22 ± 14.71	13.73 ± 5.58	82.22 ± 36.3	37.83 ± 20.54	44.44 ± 19.68	0.1306
FSE		0	5 ± 3.15	2.6 ± 2.69	7.62 ± 3.75	13.33 ± 8.63	22.5 ± 25.14	5.56 ± 4.19	4.29 ± 3.69	7.5 ± 8.45	33.45 ± 22.51	12.44 ± 9.3	58.83 ± 53.53	60.33 ± 44.82	0.1353
OS		55 ± 78.04a	29.23 ± 16.3a	10.4 ± 9.42ab	14.05 ± 6.19a	2.08 ± 1.27b	0	1.11 ± 0.82bc	0	10.75 ± 10.89ab	4.18 ± 2.6ab	0.33 ± 0.29c	23.67 ± 19.45a	2.44 ± 1.71abc	0.0474
**Incidence of pests and diseases**															
IMi	%	4.92 ± 9.81b	11.23 ± 3.85b	37.52 ± 6.21a	12.3 ± 3.03b	8.73 ± 4.01b	6.06 ± 6.94b	5.76 ± 4.63b	5.3 ± 5.25b	3.18 ± 6.94b	29.3 ± 4.18a	34.89 ± 4.63a	31.07 ± 5.67a	36.12 ± 4.63a	<0.0001
IMa		61.17 ± 11.63a	33.64 ± 4.56bc	54.68 ± 7.36a	27.82 ± 3.59 cd	48.79 ± 4.75ab	51.19 ± 8.22ab	43.74 ± 5.48ab	44.54 ± 6.22ab	51.42 ± 8.22ab	14.74 ± 4.96e	18.15 ± 5.48de	20.78 ± 6.71cde	15.56 ± 5.48de	<0.0001
**Diversity**															
RCS	Index	5.5 ± 1.74abc	6.67 ± 0.68ab	4.27 ± 1.1bc	6.52 ± 0.54ab	4.88 ± 0.71bc	5.5 ± 1.23abc	4.33 ± 0.82bc	4.93 ± 0.93bc	5.52 ± 1.23abc	3.89 ± 0.74c	3.81 ± 0.82c	7.75 ± 1a	3.98 ± 0.82c	0.0129
SWSCS		1.3 ± 0.37	1.19 ± 0.15	1.3 ± 0.23	1.37 ± 0.11	1.09 ± 0.15	1.1 ± 0.26	1.06 ± 0.17	1.15 ± 0.2	1.43 ± 0.26	1.06 ± 0.16	0.81 ± 0.17	1.59 ± 0.21	0.97 ± 0.17	0.2596
RUCS		6.25 ± 0.99	6.21 ± 0.39	5.83 ± 0.63	5.83 ± 0.31	6.5 ± 0.4	5.88 ± 0.7	6.39 ± 0.47	6.64 ± 0.53	6.23 ± 0.7	5.07 ± 0.42	5.52 ± 0.47	6.97 ± 0.57	5.51 ± 0.47	0.2911
SWUCS		1.52 ± 0.19	1.46 ± 0.08	1.48 ± 0.12	1.44 ± 0.06	1.62 ± 0.08	1.59 ± 0.14	1.63 ± 0.09	1.65 ± 0.1	1.61 ± 0.14	1.36 ± 0.08	1.5 ± 0.09	1.65 ± 0.11	1.46 ± 0.09	0.3554

Values represent the mean ± standard error. The p-value shows the differences in the capital endowment variables according to rural household types. CT: Number of cocoa trees, CS: Companion individuals, TS: Timber individuals, MS: Musaceae individuals, EFS: Food species individuals, FSE: Leguminous individuals, OS: Other companion individuals. IMi: Occurrence of *Moniliophthora roreri*, IMa: Occurrence of *Monalonion dissimulatum*, RCS: Richness of companion individuals, SWSCS: Shannon Weaver index of companion individuals, RUCS: Richness of potential uses, SWUCS: Shannon Weaver index of potential uses, CocF: Cocoa Farmers, GaCocExF: Livestock-Cocoa-Off-Farm Income, CofCocF: Coffee-Cocoa Farmers, DF: Diversified Farmers, ExCocF: External Cocoa Farmers. Light yellows highlight the lower values. Darker yellows highlight the higher values. Source: Authors’ elaboration.

The CocF and ExCocF households in Huila and CocF in Meta presented the highest density of cocoa trees, without being statistically different (p < 0.05) from each other. However, bean yield was higher in the same types of households in Meta. This department also had the lowest density of cocoa trees in the households.

Regarding the companion species, the highest density of shade canopy individuals was found in the CofCocF households in Huila, with 415 individuals ha^-1^ (Table 3). The households with the highest density of cocoa trees and the lowest density of shade canopy individuals were the CocF households in Meta. The highest density of timber trees was found in cocoa fields of households DF and GaCocExF in Caquetá, and the lowest density of this type of trees were found in households CocF in Meta. Households CofCocF, CocF and DF in Huila and ExCocF in Meta had the highest density of Musaceae in their crops, with 320, 147, 97 and 118 individuals ha^-1^, respectively. Cocoa shade food species showed no interaction (p > 0.05) between household typologies and departments (Table 3).

**Fig 1:**
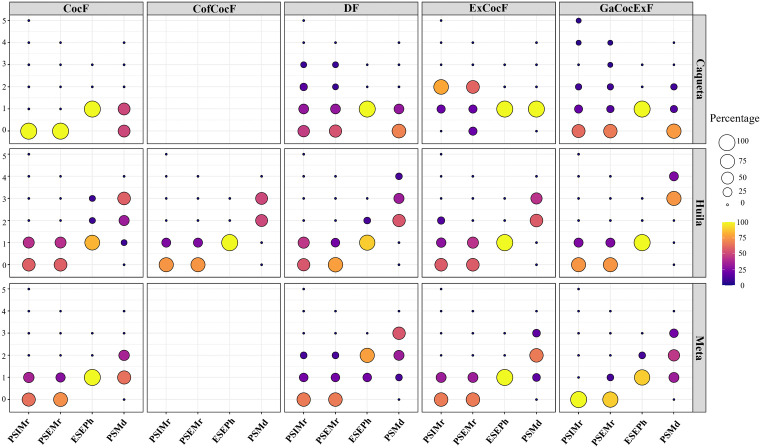
Percentage of pods with damage caused by *Monalonion dissimulatum*, *Moniliophthora roreri,* and *Phytophthora* in cocoa cultivation in rural household types in the departments of Caquetá, Huila, and Meta, Colombia. PSMd Percentage of severity of *Monalonion dissimulatum*, PSEMr Percentage of external severity *of Moniliophthora roreri*, PSIMr Percentage of internal severity of *Moniliophthora roreri*, ESEPh Percentage of external severity of *Phytophthora* Cocoa companion species other than cocoa established in the production systems, *Moniliophthora roreri*, range [0–5]: 0, No necrosis; (1) 1–20%; (2) 21–40%; (3) 41–60%; (4) 61–80%; and (5) more than 80% of the area has necrosis. *Phytophthora* spp., range [1–5]: (1) symptom-free; (2) lesion smaller than 2 mm; (3) lesion between 2 mm and 2 cm; (4) up to 25% of the pod affected; and (5) more than 25% of the pod with lesions. *Monalonion dissimulatum*, range [0–4], based on the number of stings: zero (0); 1–10 (1); 11–25 (2); 26–50 (3); and more than 50 (4), CocF: Cocoa Farmers, GaCocExF: Livestock-Cocoa-Off-Farm Income, CofCocF: Coffee-Cocoa Farmers, DF: Diversified Farmers, ExCocF: External Cocoa Farmers. Source: Authors’ elaboration.

Pest and disease severity and incidence differed among rural household types and departments. The incidence rate of *Moniliophthora roreri* was the highest in ExCocF households in Caquetá and GaCocExF, DF, ExCocF and CocF in Meta with values of 38, 36, 35, 31 and 29%, respectively (Table 3). In Meta, ExCocF and DF households also presented the highest external and internal severity of *M. roreri*, where 33 and 22% of households had, respectively, grade 1 damage. In the case of DF households, 11% of households presented external and internal severity grade 2. Although GaCocExF households presented a high incidence of this disease, the severity of internal damage was nil in the cocoa farms of these households (Fig 1).

All cocoa lots of ExCocF households in Caquetá had 100% internal damage of *M. roreri*, of these households, 20 and 80% had grade 1 and 2 damage, respectively. These households also reported the highest incidence of this disease. In this same department, 5% of GaCocExF households presented internal severity grade 5, and 4 and 19% presented internal severity grade 1; a similar pattern occurred in DF households in Caquetá, where 54% presented internal damage, of which 31, 15 and 8% of households had grade 1, 2, and 3, respectively. Finally, CofCocF and GaCocExF households in Huila presented the lowest external and internal severity.

The incidence of *Monalonium* was significantly higher (p < 0.05) in CocF and ExCocF households in Caquetá, with 61 and 55% incidence, respectively, while CocF households in Huila reported significantly lower incidence, with 15% (Table 3). Although the highest incidence of *Monalonium* was found in the CocF and ExCocF households in Caquetá, in 50% and 100% of the households the severity of damage was grade 1, and there were no households with damage in higher categories (Fig 1).

**Fig 2:**
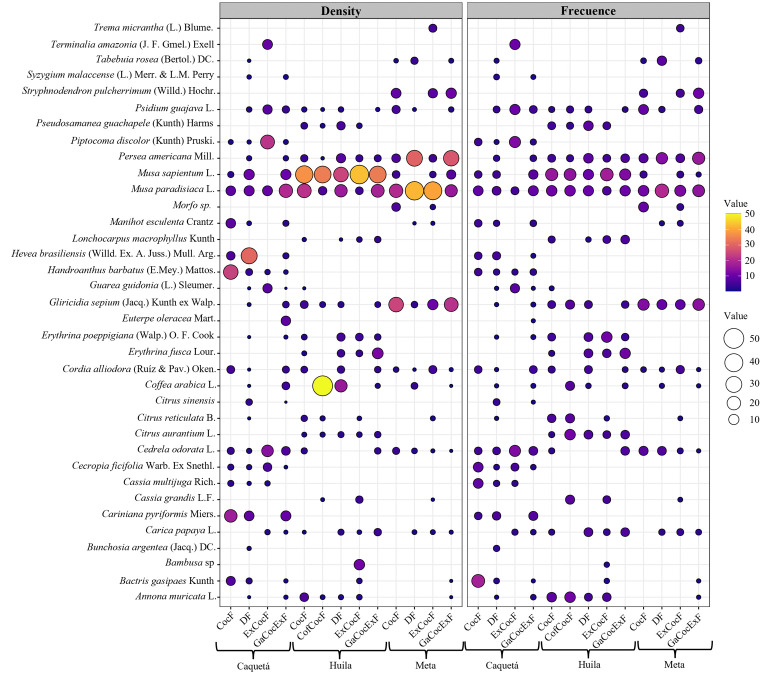
Density and frequency of shade canopy species in cocoa crop in the departments of Caquetá, Huila and Meta, Colombia. CocF: Cocoa Farmers, GaCocExF: Livestock-Cocoa-Off-Farm Income, CofCocF: Coffee-Cocoa Farmers, DF: Diversified Farmers, ExCocF: External Cocoa Farmers. Source: Authors’ elaboration.

A total of 176 species, corresponding to 117 genera and 46 families, were found in the cocoa crops of the 83 rural households. The highest species richness was found in DF and GaCocExF households in Caquetá, with 67 and 59 species, respectively, whereas the lowest number of species was found in households in CofCocF in Huila and CocF and ExCocF in Caquetá, with 14, 16, and 18 species, respectively (supplement). The species with the highest frequency and density in most households were *Musa paradisiaca* L., *Musa sapientum* L., *Persea americana* Mill. and *Gliricidia sepium* (Jacq.) Kunth ex Walp (Fig 2). *Musa sapientum* was not found in ExCocF households in Caquetá, whereas *P. americana* was not found in CocF and ExCocF households in Caquetá. In these two types of households, together with ExCocF households in Huila, no *G. sepium* (Jacq.) species were found. The density of *M. sapientum* was higher in all types of households in the department of Huila, whereas in DF and ExCocF households in Meta the high density of *M. paradisiaca* stood out (Fig 2)

### Intensity and frequency of recognized companion species

Rural households mentioned 89 cocoa companion species, grouped into a total of 17 uses (Table 4; Supplementary). The number of species found in the cocoa plots was 50.6% higher than the number recognized by the households. The most frequent uses were consumption, shade, timber, and sale (for human food).

**Table 4:** Number of species and uses mentioned, and frequency expressed as percentages of mentions by use in the types of rural households in the departments of Caquetá, Huila, and Meta, Colombia.

Department	Typology	Use	Species Mentioned	Number of uses mentioned	Number of species mentioned by use	Frequency (%) mentions use
Caquetá	GaCocExF	Consumption	49	11	17	34
		Shade			11	25
		Wood			23	15
	CocF	Consumption	11	5	7	53
		Shade			2	21
		Wood			2	11
		Future wood			3	11
	DF	Consumption	32	9	18	32
		Shade			9	28
		Wood			13	16
		Fruit sales			8	10
	ExCocF	Consumption	9	3	7	57
		Shade			3	21
		Fruit sales			3	21
Huila	GaCocExF	Shade	25	7	10	34
		Consumption			11	32
		Madera			15	19
	CocF	Shade	36	10	14	39
		Consumption			25	35
	CofCocF	Consumption	20	8	10	38
		Shade			12	32
		Fruit sales			7	20
	DF	Consumption	29	9	17	44
		Shade			16	30
		Fruit sales			11	16
	ExCocF	Consumption	27	6	17	50
		Shade			16	36
Meta	GaCocExF	Consumption	23	11	11	50
		Shade			13	27
	CocF	Consumption	12	7	8	55
		Fruit sales			5	21
		Shade			5	14
	DF	Consumption	16	7	8	45
		Shade			10	34
		Fruit sales			4	15
	ExCocF	Consumption	16	7	8	53
		Shade			7	23

The frequencies of mentions greater than 10% are presented; the complete list is provided in the supplementary. CocF: Cocoa Farmers, GaCocExF: Livestock-Cocoa-Off-Farm Income, CofCocF: Coffee-Cocoa Farmers, DF: Diversified Farmers, ExCocF: External Cocoa Farmers. Source: Authors’ elaboration

**Fig 3:**
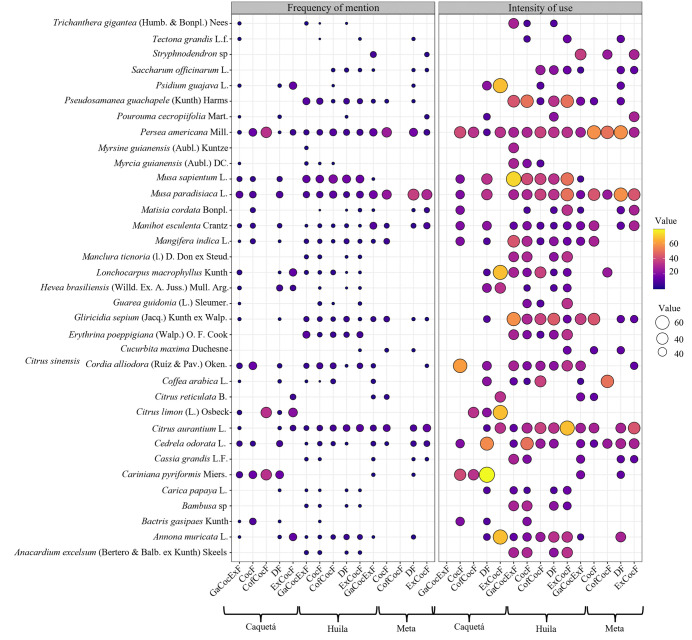
Intensity of use and frequency of mention of cocoa companion species in rural households in the departments of Caquetá, Huila, and Meta, Colombia. CocF: Cocoa Farmers, GaCocExF: Livestock-Cocoa-Off-Farm Income, CofCocF: Coffee-Cocoa Farmers, DF: Diversified Farmers, ExCocF: External Cocoa Farmers. Source: Authors’ elaboration.

The species with the highest intensity of use (IU) were *M. paradisiaca*, *P. americana*, *M. sapientum*, and *Citrus aurantium* L. In Caquetá, the highest intensity of use and frequency of mention (FM) was obtained by *Cariniana pyriformis* Miers (IU = 19%, FM = 50%) and *Citrus limon* (L.) Osbeck (IU = 15%, FM = 41%). The highest frequency of mention by household type in Caquetá was *Cordia alliodora* (Ruíz & Pav.) Oken (FM = 60%) in CocF households; *C. pyriformis* (FM = 78%) and *Cedrela odorata* L. (FM = 56%) in DF households; and *Psidium guajava* L., *Lonchocarpus macrophyllu*s Kunth, *C. limon*, and *Annona muricata* L. in ExCocF, all with a frequency of mention of 67% (Fig 3).

In Huila, the highest use intensity and mention frequency were obtained by *M. sapientum* (IU = 17%, FM = 46%), *M. paradisiaca* (IU = 10%, FM = 36%), *C. aurantium* (IU = 9%, FM = 36%), and *P. americana* (IU = 8%, FM = 33%). *Musa sapientum* was the species with the highest frequency of mention in all household types in Huila, with 71, 50, 40, 38, and 33% in GaCocExF, ExCocF, CocF, CofCocF and DF households, respectively. *Gliricidia sepium* presented a high frequency of mention in GaCocExF, DF, CofCocF, and CocF households, with 57, 44, 38, and 30%, respectively. *Persea americana* presented a high frequency of mention in CofCocF, DF, CocF and ExCocF households with 38, 33, 33, and 30%, respectively, while *C. aurantium* had a high frequency of mention in ExCocF, CofCocF, DF, and CocF households with 67, 38, 33, and 30%, respectively (Fig 3).

In Meta, the highest intensity of use and frequency of mention was recorded for *M. paradisiaca* (IU = 27%, FM = 37%), *P. americana* (IU = 16%, FM = 44%) and *C. aurantium* (IU = 11%, FM = 32%). The highest frequency of mention was in *P. americana* (FM = 57%), *M. paradisiaca* and *G. sepium* with FM of 43% in CocF, *Stryphnodendron* sp. and *G. sepium* with FM of 36% in GaCocExF, *M. paradisiaca* and *P. americana* with FM of 58% in DF, and *M. paradisiaca* and *C. aurantium* with FM of 43% in ExCocF (Fig 3).

### Relationships between agronomic crop conditions and capital for cocoa production in rural household types

**Fig 4:**
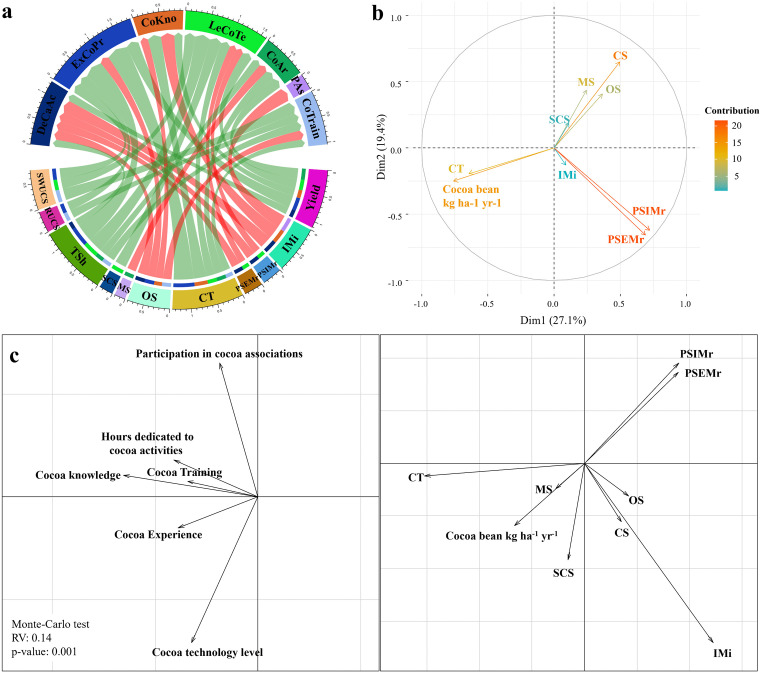
Relationships between agronomic performance and community capitals for cocoa production in rural household types in the departments of Caquetá, Huila, and Meta, Colombia. a. Significant correlations (p < 0.05) between agronomic variables and endowment variables of cocoa production b. Biplot constructed by principal component analysis using agronomic variables of cocoa production. **c.** Projection in the F1/F2 factorial plane of a Co-inertia analysis of agroforestry and production variables and capital endowment variables for cocoa production. DeCaAc: Hours dedicated to cocoa activities, ExCoPr: Cocoa Experience, CoKno: Cocoa Knowledge, LeCoTe: Cocoa technology level, CoAr: Cocoa area, PAs: Participation in cocoa associations, CoTrain: Cocoa Training, CT: Number of cocoa trees, CS: Companion individuals, TS: Timber individuals, MS: Musaceae individuals, EFS: Food species individuals, SCS: Companion shade, FSE: Leguminous individuals, OS: Other companion individuals. IMi: Occurrence of Moniliophthora roreri, IMa: Occurrence of Monalonion dissimulatum, RCS: Richness of companion individuals, SWSCS: Shannon Weaver index of companion individuals, RUCS: Richness of potential uses, SWUCS: Shannon Weaver index of potential uses. Source: Authors’ elaboration.

A significant correlation (RV = 0.14, p = 0.001) between cocoa production capital endowment variables and cocoa production variables was found (Fig 4a). Different relationships were found between the cocoa production capitals owned by households and the agronomic status of the crop (Fig 4b). Households with crops with a higher density of cocoa trees present positive correlations (p < 0.05) with years of experience (r = 0.47), knowledge (r = 0.30), cocoa area (r = 0.21), technological level (r = 0.47), and number of trainings (r = 0.16). One farmer said, “*In life you do what you know best; we knew it was cocoa. So this is the same cocoa field, but here I did the forest arrangement up to here and from here to there I planted cocoa with rubber, no more and here there is copoazú, timber, everything but in the middle of the alleys I planted pure cocoa*” (Farmer 56). In households with less area under cocoa, there is a greater density of Musaceae as part of the shade canopy of cocoa crops. Households with less time dedicated to cultivation, less knowledge, fewer years of experience and less access to training have a greater density of companion species ‘Other uses’ without high potential for commercial or food use (Fig 4b).

The incidence and severity of external and internal damage decreased significantly (p < 0.05) in households with a greater number of hours dedicated to the crop, more knowledge, higher technological level, and participation in associations (Fig 4c). One producer mentioned, “*I knew about it; I worked on it, and the crop improved. That is why you don’t see much monilia here*” (Farmer 24). Grain yield presented a positive correlation (p < 0.05) with the years of experience of the households (r = 0.28), the established area (r = 0.25), the technological level (r = 0.28), the knowledge (r = 0.28), and the number of weekly hours dedicated to the crop (r = 0.17) (Fig 4a).

## Discussion

This study demonstrates that cocoa-based agroforestry structures differ among rural household types according to livelihood strategies. The spatial structure of AFS is a basic characteristic of the dynamic processes that determine the evolution of these agroecosystems [63]. In this regard, [64] indicated that farmers constantly adapt cropping systems to meet their needs. This is influenced by different factors such as the livelihood and capital conditions of the farmer, the external environment, and the biophysical and technological factors available to them [65,66]. Hernández-Nuñez et al. [67] reported that, in the same study area, agroforestry designs (densities of cocoa trees and companion trees, shade, and types of companion species) were related to climatic variables. Proper cocoa-based AFS design generates efficient outcomes for cocoa production and other goods and services [16]. Therefore, farmers can benefit from pragmatic guidance when deciding on optimal shade levels across varying tree densities and diversity levels in AFS [20]. The management of these systems depends on the challenges and objectives of the rural household livelihood strategy [68], which determine growers’ decisions [69].

Two important variables in the design of AFS are the density of cocoa trees and shade canopy species, which determine the shade for the main species [11,13,15,16,70–75]. In this study, the density of cocoa trees was higher in households where cocoa cultivation represents the main source of livelihood (CocF and ExCocF in Huila and CocF in Meta) with 1082, 1023, and 946 individuals ha^-1^, respectively). Other researchers in the Dominican Republic have reported cocoa-based AFS ranging from 600 to 1000 individuals ha^-1^ [22]. In Cameroon, Saj et al. [3] found AFS with 1431 cocoa trees ha^-1^; in the same country, Abada et al. [12] found plantations with a mean density of 843 cocoa trees ha^-1^, but more than 40% of the plantations had a density greater than 1000. However, FEDECACAO technical guidelines recommend a density of 600−700 cocoa trees ha^-1^ [76], which, in our study, were the lowest densities and were present in ExCocF household types in Meta. The divergence of AFS designs in relation to national production guidelines may be due to the fact that guideline construction rarely involves the participation of producers [77].

The density of shade canopy species was different between typologies, being higher in the CofCocF households in Huila with 415 individuals ha^-1^, which presented a high density of Musaceae, and lower in the ExCocF households in Caquetá and in CocF households in Meta. Previous research has identified several factors that determine the density and type of shade canopy species [40,78]. The diversity of crop species generates a range of phenological, morphological, and physiological traits, whose combination influences the agroecological functioning of AFS through facilitation or competition effects [64]. One factor of importance when establishing shade canopy species is regulating shade for cocoa, as it directly influences the agronomic yield of cocoa beans [13,49,50,53,75]. In some cases, farmers have found it necessary to remove forest tree species to achieve higher bean yields [64,79]. Moreover, authors such as Trebissou et al. [80] indicate that competition between companion trees and cocoa trees increases over time. Also, Hernández-Núñez et al. [21] concluded that proper management of shade canopy species can generate high cocoa bean yields and different environmental and economic services.

Defining the percentage of shade for cocoa is accompanied by different reasons that the producer may have to determine the type of species and the quantity to plant or leave during the cocoa planting process. Among them are the various environmental, economic, and food security services that can be generated [17]. Some of these are: a) the improvement of soil conditions [79], b) biodiversity conservation and the generation of ecosystem services [11,17,81,82], c) adaptive capacity to climate variability and change [20,22,83–85], d) food production that diversifies the diet, and e) economic income for farmers [17]. Although environmental services are of global interest, Sanial et al. [69] indicate that there are socioeconomic variables that are more determinant than environmental variables in making decisions on the association of trees with cocoa. This is consistent with the results of this study, as the type of rural household, which is grouped by livelihood strategies, has a significant influence on the design of cocoa-based AFS.

In these plots, 176 cocoa companion species were found, of which only 89 were mentioned by the farmers. Similar results were found by Ndo et al. [63], who identified a high inconsistency between the observed spatial structures of the trees in AFS and those declared by the farmers. Additionally, Sonwa et al. [86] found a greater number of plants associated with cocoa than those mentioned by the producers, indicating that most recognized plants correspond to species of high interest according to their use. This suggests that there is more unplanned aboveground plant diversity in cocoa plantations [72]. In Africa, Indonesia, and Latin America, cocoa-based AFS have been used as alternatives to improve the welfare of local people by adding commercial value and elements associated with other plant species, such as fruit trees (e.g., orange, avocado, pitahaya, and black pepper) and others for timber purposes (e.g., cedar, mahogany) [20].

The diversity of species mentioned by farmers varies according to the livelihood strategy of the household. This may be because the choice of shade species is associated with advantages and disadvantages for development on a cocoa farm, for the diversification of income sources, and for the maintenance of biological diversity [78]. This is reaffirmed by Jagoret et al. [87], who indicate that farmers distinguish these species according to their positive or negative effects on cocoa trees. Consequently, the diversity of shade canopy species varies between communities and countries. In research conducted in Africa, indigenous and non-indigenous ethnocultural groups cited 174 species associated with cocoa trees [78], but in Cameroon, an average of 21 species were associated with cocoa within AFS [86], although farmers tended to use only seven plant species (four fruiting species and three non-fruiting species). This diversification has been identified as an important agroecological strategy for rural development [88].

The species mentioned by farmers in this study were grouped into 17 uses, the most frequent being consumption, shade, timber, and sale (for human food). The diversity and composition of cocoa-based AFS can benefit cocoa growth and help meet household food requirements, provide additional income, and supply material for construction [14,17,20,72,77,89]. Therefore, farmers’ uses of species are linked to their needs and knowledge of the environment [77]. In some cases, the contribution of the agroforestry product mix to household profit is similar to or higher than that of cocoa beans [14]. For example, Notaro et al. [23] mentioned that after 12 years, cocoa production generated income of US$590 to US$650 ha^-1^ yr^-1^, while associated timber trees generated income of US$1800 to US$2300 ha^-1^ yr^-1^.

In Togo, Africa, Guelly et al. [78] reported that shade canopy species provide 18 services, the most relevant being shade provided to cocoa trees (98%), contribution to household food and nutritional security (92%), and diversification of household income sources (79%). In Côte d’Ivoire, tree species were selected mainly to provide shade for cocoa and to produce products that generate income from sales [66]. In Mexico, Zequeira-Larios et al. [20] reported that 55% of species were used for food, 31% for timber, 24% for other uses, 14% for medicine, and 12% for shade for cocoa. In the same country, Soto-Pinto et al. [90] reported that of the 48 useful species recorded in cocoa systems, 23 were used as food, 15 of which were native. The multifunctionality of cocoa-based AFS corresponds to a complexity established by farmers and largely explains the high level of agrobiodiversity in these complex systems [77]. Authors such as Anglaaere et al. [79] have reported that farmers value companion species for their lumber quality, fruit, or medicinal value. These results indicate the urgent need to recognize the non-monetary value of local food, agrobiodiversity, and local knowledge in Latin America [90].

The species with the highest intensity of use were *Musa paradisiaca* L., *Persea americana* Mill., *Musa sapientum* L., and *Citrus aurantium* L. This coincides with reports in different investigations, for example, Jagoret et al. [77] in Cameroon, Asante et al. [74] in Ghana, Soto-Pinto et al. [90] in Mexico, and Hernández-Núñez et al. [21] in Colombia reported that in cocoa plantations, *Persea americana* Mill. (fruiting) was one of the species other than cocoa with the highest use values. Sanial et al. [69] and Zequeira-Larios et al. [20] estimated a high frequency in cocoa-based AFS of species of the genus Citrus (fruit). These results were similar in Huila and Meta, where the most important species for the families were *Musa sapientum* L., *Musa paradisiaca* L., *Citrus aurantium* L. and *Persea americana* Mill. In line with these reports, Atangana et al. [66] indicate that in cocoa-based AFS, fruit tree species were the most planted. These types of species play an important role in food supply and generate opportunities, as they can give rise to local, informal, and specialized fruit value chains [69].

In Caquetá, a high density of timber trees was found in the DF and GaCocExF households, and one of the most important species for the families was *Cordia alliodora* (Ruíz & Pav.) Oken. In Costa Rica, Rousseau et al. [91], and in Mexico, Zequeira-Larios et al. [21] found that this same species was one of the most common in their research. Likewise, Atangana et al. [67] reported that timber species were mostly conserved at the time of logging for cocoa planting. In Cameroon, they found that the value of timber is not necessarily a priority for farmers when they decide to conserve forest species in their cocoa plantations [12]; however, this changes according to local conditions, for example, in the department of Caquetá, there is a forest vocation. This is confirmed by Sonwa et al. [87], who indicated that the level of market access influences the types of species managed within cocoa AFS.

A correlation was found between capital endowment variables for cocoa production and cocoa production variables. In this regard, Scudder et al. [2] indicate that labor and capital costs represent an important part of any production system. Cerda et al. [14] mentioned that the relationships between socioeconomic indicators and agroforestry product yields are determined by the AFS typology. In the case of the present study, cocoa bean yields were determined by the rural household typology according to the livelihood strategy.

Assets such as knowledge, experience in cultivation, technological level, and dedication to cultivation have an impact on agronomic performance. Accordingly, Notaro et al. [64] have mentioned that agricultural innovations can originate from the farmers themselves, from public or private extension services, from technical institutes, or from research. In addition, traditional AFS have historically sustained peasant communities, where local agrobiodiversity, indigenous practices, and knowledge play an important role [90]. An example of this is that carrying out pruning activities requires many resources and knowledge [72]. Additionally, the technological level to achieve different cocoa yields requires labor and financial capital [2].

The incidence and severity of external and internal damage of monilia decrease in households with greater dedication to the crop, knowledge, technological level, and participation in associations. Scudder et al. [2] state that a consequence of low cocoa yields was the increase of pests and diseases. Amon-Armah et al. [92] have mentioned that lack of knowledge of farmers can lead to poor management of insect pests and, therefore, to economic losses. Additionally, Denkyirah et al. [93] found that cocoa farmers’ decision to use pesticides in cocoa production was influenced by farmer’s gender, farmer’s age, farmer’s educational level, years of farming experience, access to extension services, among other factors. Therefore, more training is needed to improve farmers’ knowledge of the biology and behavior of key pests in cocoa for effective management [92]. It is important to comprehend that there is an intermediate stage in the process of technological adoption and improvement, where farmer characteristics and economic variables affect adoption indirectly by influencing knowledge, attitudes, and perceptions, affecting farmers’ decisions to adopt or not adopt an innovation [94].

Bean yields were positively correlated with the households’ years of experience, established area, technological level, knowledge, and number of hours per week dedicated to the crop. Accordingly, it has been determined that many smallholder cocoa farmers experience low crop yields due to generally poor management practices, pests, diseases, and low soil fertility [2]. This is related to the fact that management practices are influenced by certain farmer characteristics such as age, professional experience, or educational level [78]. This is reaffirmed by Jagoret et al. [77], who concluded that technical innovations designed to improve cocoa AFS must take into account farmers’ knowledge.

The typologies CofCocF and ExCocF in Huila, and DF in Meta had high yields and species abundance. This agrees with the statement of Ndo et al. [63], who mention that appropriate spatial structures can be adopted, provided that farmers are well informed and trained in modern agroforestry techniques. Therefore, improved management can increase smallholder cocoa yields and improve the procurement of other products [21]. However, despite the potential benefits of AFS, its implementation and management are challenging [72]. Therefore, significant efforts would be required to boost access to information and training for cocoa farmers to improve cocoa farmers’ techniques [95], and it is important to keep in mind that higher workloads with increased system complexity (inadequate designs) have led farmers to abandon agroforestry plots [72].

### Implications of the study and policy directions

Our results show that the structure and management of cocoa-based AFS are linked to the livelihood strategies and capital endowments of rural households. This has relevant implications for both the formulation of public policies and the design of technical programs to support the promotion and strengthening of cocoa farming. We highlight the need to move from generalist approaches to differentiated schemes tailored to the conditions of the territory. The results indicate that it is necessary to reformulate the technical guidelines on planting densities and companion species in AFS, incorporating local knowledge and the priorities of producers. The divergence found between official recommendations, such as those of FEDECACAO, and actual practices responds, in part, to empirical adaptation processes based on farmers’ experiences, capacities and limitations. Therefore, policies should be co-constructed with producers, ensuring their relevance, adaptation, and sustainability. We consider it necessary that rural policies promote multifunctional agroforestry systems, which not only ensure acceptable cocoa yields but also contribute to food security, economic diversification, and climate resilience of families under agroecological approaches. Considering that higher yields and lower incidences of diseases are related to factors such as dedication to the crop, knowledge, experience, and participation in associations it is necessary to strengthen training and cooperation processes, prioritizing the types of households with lower levels of human and social capital. Finally, the differences observed between departments (Huila, Meta, and Caquetá) indicate the relevance of developing regionalized agroforestry policies that consider local conditions.

## Conclusion and recommendations

The agronomic (pest and disease affectation and yield) and agroforestry design (tree structure, floristic composition, and density of cocoa trees) conditions in cocoa-based AFS different between the combination of departments (different geographic zones) and rural household types. This design depends on the objectives that rural households have, the capacities they have (capital endowment), the environment, and other contextual conditions. These define the commercial processes and opportunities, which influence the selection of cocoa companion plants.

It was found that the density of cocoa trees is higher in households where the most important livelihood is cocoa (CocF in Huila and Meta), and this type of household in Meta presented the lowest density of companion species. Likewise, ExCocF households in Meta had a low density of cocoa trees (676 individuals ha^-1^). The density of companion species also showed interaction between the type of rural household and the department, being higher in CofCocF households in Huila, with 415 individuals ha^-1^, where the crop was in AFS with a high density of Musaceae. It was found that rural households only recognize and use less than 50% of the species they have as companion species in cocoa crops. Seventeen uses were mentioned for these species, the most frequent being consumption, shade, timber, and sale (human food species).

The variables of capital endowment for cocoa production and cocoa production variables were correlated. Households with a greater density of cocoa trees, more years of experience in cocoa cultivation, better technical knowledge, larger planted areas, and higher technological levels showed better agronomic performance. The incidence and severity of external and internal Monilia damage decreased significantly in households with more hours dedicated to cultivation, knowledge, technological level, and participation in associations. Finally, the results indicated that households with more years of experience in cocoa cultivation, higher technological level for production, better knowledge, and more hours per week dedicated to cultivation had higher cocoa bean yields. Therefore, the results allow us to conclude that the management and efficiency of cocoa-based AFS depend on the articulation of social processes and the design of the AFS.

## Supporting information

S1 File Database. (XLSX)

S2 File Supplementary material. (DOCX)
