# Supplementary material for: Agronomic performance of cocoa production type systems in Colombia
Source: PLoS One. 2025 Dec 3;20(12):e0337624. doi: 10.1371/journal.pone.0337624 (PMC12674545; doi:10.1371/journal.pone.0337624)
Supplement: S2 File — (DOCX) [file pone.0337624.s002.docx]

**Supplementary material**

**Agronomic performance of cocoa production type systems in Colombia**

Héctor Eduardo Hernández-Nuñez^1,2,3*^, Juan Carlos Suárez^2,4^, Hernán J. Andrade^3^, Angie Paola Bernal Núñez^4^, David Ricardo Gutiérrez^4^, Gustavo Adolfo Gutiérrez^1,2,4^, Isabel Gutiérrez-Montes^5^, V. Ernesto Méndez ^6^, Fernando Casanoves^1,2,5^

^1^ Programa de Doctorado en Ciencias Naturales y Desarrollo Sustentable, Facultad de Ciencias Agropecuarias, Universidad de la Amazonia, Florencia, Caquetá, Colombia

^2^Centro de Investigaciones Amazónicas CIMAZ Macagual Cesar Augusto Estrada González, Grupo de Investigaciones Agroecosistemas y Conservación en Bosques Amazónicos- GAIA

^3^Programa de Doctorado en Ciencias Agrarias, Facultad de Ingeniería Agronómica, Universidad del Tolima, Ibagué, Tolima, Colombia

^4^Programa de Ingeniería Agroecológica, Facultad de Ingeniería, Universidad de la Amazonia, Florencia, Caquetá, Colombia

^5^CATIE – Centro Agronómico Tropical de Investigación y Enseñanza. Turrialba, 30501, Costa Rica

^6^Institute for Agroecology (IfA), University of Vermont, Burlington, VT, USA

* Corresponding author: h.hernandez@udla.edu.co

*Table 1. Variables used to assess community capitals at the cocoa production level.*

| Capital | Variable | Unit | Description |
| --- | --- | --- | --- |
| Cultural Capital | a. Experience in cocoa cultivation | Years | Total number of years the person has been involved in cocoa farming. |
|  | b. Dedication to cocoa farming activities | Hours/week | Weekly hours dedicated exclusively to cocoa farming. |
|  | c. Identity with cocoa cultivation | Categorical | Degree of identification or sense of belonging to cocoa farming. |
|  | d. Motivations when planting cocoa | Number | Main reasons or motivations for planting cocoa. |
| Natural Capital | e. Cocoa area | Hectares (ha) | Total land area allocated to cocoa cultivation. |
| Human Capital | f. Cocoa trainings | Number | Number of trainings received related to cocoa cultivation. |
|  | g. Cocoa knowledge | Scale 1–5 (5 = highest) | Self-assessed level of knowledge about cocoa farming practices. |
| Built Capital | h. Technological level for cocoa cultivation | Categorical (Low/Med/High) | Level of technological adoption in cocoa production. |
| Social and Political Capital | i. Participation in cocoa associations | Yes/No | Indicates whether the farmer is a member of a cocoa association. |
| Financial Capital | j. Cocoa dry bean production | Kilograms/household/year | Annual quantity of dry cocoa beans produced per household. |
|  | k. Annual income from cocoa | Dollars/year | Annual income derived exclusively from cocoa production. |

*Table 2. Frequency of shade canopy species in cocoa crop in the departments of Caquetá, Huila and Meta, Colombia.*

| **Typology** | **Department** | **Number of species** |
| --- | --- | --- |
| Livestock-Cocoa-Off-Farm Income | Caquetá | 59 |
|  | Huila | 23 |
|  | Meta | 33 |
| External Cocoa Farmers | Caquetá | 18 |
|  | Huila | 20 |
|  | Meta | 32 |
| Diversified Farmers | Caquetá | 67 |
|  | Huila | 21 |
|  | Meta | 22 |
| Coffee-Cocoa Farmers | Huila | 14 |
| Cocoa Farmers | Caquetá | 16 |
|  | Huila | 27 |
|  | Meta | 23 |

Table 3. Species and uses mentioned in the types of rural households in the departments of Caquetá, Huila and Meta, Colombia.

| **Typology** | **Department** | **Scientific name** | **Use** |
| --- | --- | --- | --- |
| CocF | Caqueta | Apeiba membranacea | Shade |
|  |  | Bactris gasipaes Kunth | Human Food |
|  |  | Cariniana pyriformis Miers. | Shade |
|  |  |  | Wood reserve |
|  |  | Cedrela odorata L. | Wood |
|  |  | Cordia alliodora (Ruíz & Pav.) Oken. | Shade |
|  |  |  | Wood |
|  |  |  | Wood reserve |
|  |  | Mangifera indica L. | Human Food |
|  |  | Manihot esculenta Crantz | Human Food |
|  |  | Matisia cordata Bonpl. | Human Food |
|  |  | Musa paradisiaca L. | Human Food |
|  |  | Musa sapientum L. | Human Food |
|  |  | Persea americana Mill. | Human Food |
|  |  |  | Sales |
|  | Huila | Anacardium excelsum (Bertero & Balb. ex Kunth) Skeels | Humidity Conservation |
|  |  |  | Shade |
|  |  |  | Wood |
|  |  | Annona muricata L. | Human Food |
|  |  |  | Shade |
|  |  | Bactris gasipaes Kunth | Human Food |
|  |  |  | Sales |
|  |  | Bambusa sp | Live fence |
|  |  |  | Reforestation |
|  |  |  | Soil conservation |
|  |  | Carica papaya L. | Human Food |
|  |  | Carludovica palmata Ruiz & Pav | Shade |
|  |  | Cassia grandis L.F. | Firewood |
|  |  |  | Shade |
|  |  | Cecropia peltata L. | Reforestation |
|  |  |  | Shade |
|  |  | Cedrela odorata L. | Human Food |
|  |  |  | Humidity Conservation |
|  |  |  | Sales |
|  |  |  | Shade |
|  |  |  | Wood |
|  |  | Ceiba pentandra (L.) Gaertn. | Shade |
|  |  | Citrus aurantium L. | Human Food |
|  |  |  | Sales |
|  |  |  | Shade |
|  |  | Coffea arabica L. | Human Food |
|  |  | Cordia alliodora (Ruíz & Pav.) Oken. | Shade |
|  |  |  | Wood |
|  |  | Croton lechleri | Shade |
|  |  | Erythrina poeppigiana (Walp.) O. F. Cook | Firewood |
|  |  |  | Shade |
|  |  | Eucalyptus sp. | Wood |
|  |  | Eugenia stipitata McVaugh | Human Food |
|  |  | Ficus americana Aubl. subsp. americana | Shade |
|  |  | Gliricidia sepium (Jacq.) Kunth ex Walp. | Humidity Conservation |
|  |  |  | Shade |
|  |  |  | Soil conservation |
|  |  | Guarea guidonia (L.) Sleumer. | Shade |
|  |  |  | Wood |
|  |  | Hevea brasiliensis (Willd. ex A. Juss.) Müll. Arg. | Shade |
|  |  | Lonchocarpus macrophyllus Kunth | Firewood |
|  |  |  | Shade |
|  |  | Maclura tinctoria (L.) D. Don ex Steud. | Live fence |
|  |  |  | Shade |
|  |  |  | Wood |
|  |  | Mammea americana L. | Human Food |
|  |  | Mangifera indica L. | Human Food |
|  |  |  | Sales |
|  |  |  | Shade |
|  |  | Manihot esculenta Crantz | Human Food |
|  |  |  | Shade |
|  |  | Matisia cordata Bonpl. | Human Food |
|  |  | Musa paradisiaca L. | Human Food |
|  |  |  | Sales |
|  |  |  | Shade |
|  |  | Musa sapientum L. | Animal food |
|  |  |  | Human Food |
|  |  |  | Sales |
|  |  |  | Shade |
|  |  | Myrcia guianensis (Aubl.) DC. | Shade |
|  |  |  | Wood |
|  |  | Ochroma pyramidale (Cav. Ex Lam.) Urb. | Shade |
|  |  | Persea americana Mill. | Human Food |
|  |  |  | Sales |
|  |  |  | Shade |
|  |  | Pseudosamanea guachapele (Kunth) Harms | Humidity Conservation |
|  |  |  | Live fence |
|  |  |  | Sales |
|  |  |  | Shade |
|  |  |  | Wood |
|  |  | Tectona grandis L.f. | Sales |
|  |  | Trichanthera gigantea (Humb. & Bonpl.) Nees | Live fence |
|  |  | Zygia longifolia (Willd.) Britton & Rose | Soil conservation |
|  | Meta | Cedrela odorata L. | Wood |
|  |  | Citrus aurantium L. | Human Food |
|  |  |  | Sales |
|  |  | Citrus reticulata B. | Human Food |
|  |  | Cocos nucifera L. | Human Food |
|  |  | Cucurbita maxima Duchesne | Human Food |
|  |  | Gliricidia sepium (Jacq.) Kunth ex Walp. | Firewood |
|  |  |  | Medical |
|  |  |  | Shade |
|  |  | Leucaena leucocephala (Lam.) de Wit | Shade |
|  |  | Mangifera indica L. | Human Food |
|  |  |  | Sales |
|  |  | Manihot esculenta Crantz | Human Food |
|  |  |  | Sales |
|  |  | Musa paradisiaca L. | Human Food |
|  |  |  | Sales |
|  |  |  | Shade |
|  |  | Persea americana Mill. | Animal food |
|  |  |  | Human Food |
|  |  |  | Sales |
|  |  |  | Shade |
|  |  | Pseudosamanea guachapele (Kunth) Harms | Shade |
| CoFCocF | Caqueta | Cariniana pyriformis Miers. | Wood |
|  |  | Citrus limon (L.) Osbeck | Shade |
|  |  | Persea americana Mill. | Human Food |
|  | Huila | Annona muricata L. | Human Food |
|  |  |  | Sales |
|  |  | Cedrela odorata L. | Shade |
|  |  |  | Wood |
|  |  | Citrus aurantium L. | Human Food |
|  |  |  | Sales |
|  |  |  | Shade |
|  |  | Coffea arabica L. | Human Food |
|  |  |  | Sales |
|  |  |  | Soil conservation |
|  |  | Cordia alliodora (Ruíz & Pav.) Oken. | Shade |
|  |  |  | Wood |
|  |  | Cupania americana Linneo. | Live fence |
|  |  |  | Shade |
|  |  | Erythrina poeppigiana (Walp.) O. F. Cook | Shade |
|  |  | Eugenia stipitata McVaugh | Sales |
|  |  | Gliricidia sepium (Jacq.) Kunth ex Walp. | Animal food |
|  |  |  | Live fence |
|  |  |  | Shade |
|  |  | Guarea guidonia (L.) Sleumer. | Shade |
|  |  | Lonchocarpus macrophyllus Kunth | Firewood |
|  |  |  | Shade |
|  |  |  | Wood |
|  |  | Mangifera indica L. | Human Food |
|  |  | Manihot esculenta Crantz | Human Food |
|  |  | Musa paradisiaca L. | Human Food |
|  |  |  | Sales |
|  |  |  | Shade |
|  |  | Musa sapientum L. | Human Food |
|  |  |  | Sales |
|  |  |  | Shade |
|  |  | Myrcia guianensis (Aubl.) DC. | Wood |
|  |  | Persea americana Mill. | Human Food |
|  |  |  | Sales |
|  |  |  | Shade |
|  |  | Pseudosamanea guachapele (Kunth) Harms | Shade |
|  |  | Psidium guajava L. | Human Food |
|  |  | Saccharum officinarum L. | Human Food |
|  |  |  | Soil conservation |
|  | Meta | Cedrela odorata L. | Shade |
|  |  | Coffea arabica L. | Human Food |
|  |  |  | Sales |
|  |  | Lonchocarpus macrophyllus Kunth | Firewood |
|  |  | Musa paradisiaca L. | Human Food |
|  |  | Persea americana Mill. | Human Food |
|  |  |  | Shade |
|  |  | Stryphnodendron sp | Shade |
| DF | Caqueta | Acrocarpus fraxinifolius Arn. | Shade |
|  |  |  | Wood |
|  |  | Ananas comosus L. | Human Food |
|  |  |  | Sales |
|  |  | Annona muricata L. | Human Food |
|  |  | Baccharis latifolia (Ruíz & Pav.) Pers. | Reforestation |
|  |  |  | Wood |
|  |  | Bactris gasipaes Kunth | Human Food |
|  |  | Capparis amplissima Lam. | Shade |
|  |  | Carica papaya L. | Human Food |
|  |  | Cariniana pyriformis Miers. | Firewood |
|  |  |  | Ornamental |
|  |  |  | Reforestation |
|  |  |  | Shade |
|  |  |  | Soil conservation |
|  |  |  | Wood |
|  |  |  | Wood reserve |
|  |  | Cedrela odorata L. | Ornamental |
|  |  |  | Reforestation |
|  |  |  | Shade |
|  |  |  | Soil conservation |
|  |  |  | Wood |
|  |  | Cedrelinga catanaeformis Ducke | Reforestation |
|  |  |  | Shade |
|  |  |  | Wood |
|  |  | Citrus aurantium L. | Human Food |
|  |  | Citrus limon (L.) Osbeck | Human Food |
|  |  |  | Sales |
|  |  | Coffea arabica L. | Human Food |
|  |  |  | Sales |
|  |  | Cordia alliodora (Ruíz & Pav.) Oken. | Shade |
|  |  |  | Wood |
|  |  | Eugenia stipitata McVaugh | Human Food |
|  |  | Gliricidia sepium (Jacq.) Kunth ex Walp. | Soil conservation |
|  |  | Hevea brasiliensis (Willd. ex A. Juss.) Müll. Arg. | Sales |
|  |  |  | Shade |
|  |  | Lonchocarpus macrophyllus Kunth | Human Food |
|  |  | Mangifera indica L. | Human Food |
|  |  | Manihot esculenta Crantz | Human Food |
|  |  |  | Sales |
|  |  | Minquartia guianensis Aubl. | Shade |
|  |  | Musa paradisiaca L. | Human Food |
|  |  |  | Sales |
|  |  |  | Shade |
|  |  | Musa sapientum L. | Human Food |
|  |  |  | Sales |
|  |  |  | Shade |
|  |  | Nectandra acuminata (Nees & C.Mart.) J.F.Macbr. | Reforestation |
|  |  |  | Wood |
|  |  | Nectandra acutifolia (Ruiz & Pav.) Mez. | Reforestation |
|  |  |  | Shade |
|  |  |  | Wood |
|  |  | Persea americana Mill. | Human Food |
|  |  | Pourouma cecropiifolia Mart. | Human Food |
|  |  | Psidium guajava L. | Human Food |
|  |  |  | Shade |
|  |  | Solanum sessiliflorum Dunal | Human Food |
|  |  | Terminalia amazonia (J. F. Gmel.) Exell | Shade |
|  |  | Vochysia braceliniae | Reforestation |
|  |  |  | Wood |
|  |  | Zea mays L. | Human Food |
|  |  |  | Sales |
|  | Huila | Anacardium excelsum (Bertero & Balb. ex Kunth) Skeels | Live fence |
|  |  |  | Shade |
|  |  | Annona muricata L. | Human Food |
|  |  |  | Sales |
|  |  |  | Shade |
|  |  | Bambusa sp | Ornamental |
|  |  |  | Soil conservation |
|  |  | Carica papaya L. | Human Food |
|  |  | Cedrela odorata L. | Shade |
|  |  |  | Wood |
|  |  | Citrus aurantium L. | Human Food |
|  |  |  | Sales |
|  |  |  | Shade |
|  |  | Cocos nucifera L. | Human Food |
|  |  | Cordia alliodora (Ruíz & Pav.) Oken. | Shade |
|  |  |  | Wood |
|  |  | Erythrina poeppigiana (Walp.) O. F. Cook | Shade |
|  |  |  | Wood |
|  |  | Eugenia stipitata McVaugh | Human Food |
|  |  | Euterpe oleracea Mart. | Human Food |
|  |  |  | Sales |
|  |  | Gliricidia sepium (Jacq.) Kunth ex Walp. | Animal food |
|  |  |  | Live fence |
|  |  |  | Shade |
|  |  |  | Wood |
|  |  | Hevea brasiliensis (Willd. ex A. Juss.) Müll. Arg. | Shade |
|  |  | Jatropha curcas | Shade |
|  |  |  | Wood |
|  |  | Lonchocarpus macrophyllus Kunth | Human Food |
|  |  | Maclura tinctoria (L.) D. Don ex Steud. | Shade |
|  |  |  | Wood |
|  |  | Mammea americana L. | Human Food |
|  |  |  | Sales |
|  |  |  | Shade |
|  |  | Mangifera indica L. | Human Food |
|  |  |  | Shade |
|  |  | Manihot esculenta Crantz | Human Food |
|  |  | Matisia cordata Bonpl. | Human Food |
|  |  | Musa paradisiaca L. | Human Food |
|  |  |  | Sales |
|  |  |  | Shade |
|  |  | Musa sapientum L. | Human Food |
|  |  |  | Sales |
|  |  |  | Shade |
|  |  | Passiflora maliformis L. | Sales |
|  |  | Persea americana Mill. | Human Food |
|  |  |  | Sales |
|  |  |  | Shade |
|  |  | Pourouma cecropiifolia Mart. | Human Food |
|  |  |  | Sales |
|  |  | Pouteria caimito (Ruíz & Pav.) Radlk. | Human Food |
|  |  |  | Sales |
|  |  | Pseudosamanea guachapele (Kunth) Harms | Live fence |
|  |  |  | Shade |
|  |  |  | Wood |
|  |  | Saccharum officinarum L. | Human Food |
|  |  |  | Sales |
|  |  | Trichanthera gigantea (Humb. & Bonpl.) Nees | Reforestation |
|  | Meta | Acacia mangium Willd. | Shade |
|  |  |  | Windbreak barrier |
|  |  | Annona muricata L. | Human Food |
|  |  |  | Shade |
|  |  | Cariniana pyriformis Miers. | Shade |
|  |  | Cassia grandis L.F. | Shade |
|  |  | Cedrela odorata L. | Shade |
|  |  |  | Wood reserve |
|  |  | Citrus aurantium L. | Human Food |
|  |  |  | Sales |
|  |  | Cucurbita maxima Duchesne | Human Food |
|  |  | Gliricidia sepium (Jacq.) Kunth ex Walp. | Shade |
|  |  | Manihot esculenta Crantz | Human Food |
|  |  | Matisia cordata Bonpl. | Sales |
|  |  | Musa paradisiaca L. | Animal food |
|  |  |  | Human Food |
|  |  |  | Sales |
|  |  |  | Shade |
|  |  | Persea americana Mill. | Human Food |
|  |  |  | Sales |
|  |  |  | Shade |
|  |  |  | Soil conservation |
|  |  | Pseudosamanea guachapele (Kunth) Harms | Shade |
|  |  | Psidium guajava L. | Human Food |
|  |  | Saccharum officinarum L. | Human Food |
|  |  | Tectona grandis L.f. | Shade |
| ExCocF | Caqueta | Annona muricata L. | Human Food |
|  |  |  | Sales |
|  |  | Bertholletia excelsa Bonpl. | Shade |
|  |  | Citrus aurantium L. | Human Food |
|  |  | Citrus limon (L.) Osbeck | Human Food |
|  |  |  | Sales |
|  |  | Citrus reticulata B. | Human Food |
|  |  | Hevea brasiliensis (Willd. ex A. Juss.) Müll. Arg. | Shade |
|  |  | Lonchocarpus macrophyllus Kunth | Human Food |
|  |  |  | Shade |
|  |  | Persea americana Mill. | Human Food |
|  |  | Psidium guajava L. | Human Food |
|  |  |  | Sales |
|  | Huila | Anacardium excelsum (Bertero & Balb. ex Kunth) Skeels | Shade |
|  |  |  | Wood |
|  |  | Ananas comosus L. | Human Food |
|  |  | Annona cherimola Mill. | Human Food |
|  |  | Annona muricata L. | Human Food |
|  |  |  | Shade |
|  |  | Bambusa sp | Live fence |
|  |  | Carica papaya L. | Human Food |
|  |  | Citrus aurantium L. | Fertilization |
|  |  |  | Human Food |
|  |  |  | Sales |
|  |  |  | Shade |
|  |  | Cordia alliodora (Ruíz & Pav.) Oken. | Shade |
|  |  |  | Wood |
|  |  | Cucurbita maxima Duchesne | Human Food |
|  |  | Erythrina poeppigiana (Walp.) O. F. Cook | Shade |
|  |  |  | Wood |
|  |  | Gliricidia sepium (Jacq.) Kunth ex Walp. | Shade |
|  |  | Gmelina arborea Roxb. ex Sm. | Shade |
|  |  | Guarea guidonia (L.) Sleumer. | Human Food |
|  |  |  | Shade |
|  |  | Hevea brasiliensis (Willd. ex A. Juss.) Müll. Arg. | Shade |
|  |  | Lonchocarpus macrophyllus Kunth | Human Food |
|  |  | Maclura tinctoria (L.) D. Don ex Steud. | Shade |
|  |  |  | Wood |
|  |  | Mangifera indica L. | Human Food |
|  |  | Manihot esculenta Crantz | Human Food |
|  |  | Matisia cordata Bonpl. | Human Food |
|  |  |  | Shade |
|  |  | Musa paradisiaca L. | Human Food |
|  |  |  | Sales |
|  |  |  | Shade |
|  |  | Musa sapientum L. | Human Food |
|  |  |  | Sales |
|  |  |  | Shade |
|  |  | Passiflora edulis Sims | Human Food |
|  |  | Passiflora maliformis L. | Human Food |
|  |  | Persea americana Mill. | Human Food |
|  |  |  | Shade |
|  |  | Pseudosamanea guachapele (Kunth) Harms | Live fence |
|  |  |  | Shade |
|  |  |  | Wood |
|  |  | Saccharum officinarum L. | Human Food |
|  |  | Tectona grandis L.f. | Shade |
|  | Meta | Bauhinia tarapotensis Benth. | Firewood |
|  |  | Cassia grandis L.F. | Shade |
|  |  | Cedrela odorata L. | Shade |
|  |  |  | Wood |
|  |  | Citrus aurantium L. | Human Food |
|  |  |  | Sales |
|  |  |  | Shade |
|  |  | Cocos nucifera L. | Shade |
|  |  | Cordia alliodora (Ruíz & Pav.) Oken. | Wood |
|  |  | Gliricidia sepium (Jacq.) Kunth ex Walp. | Medical |
|  |  | Manihot esculenta Crantz | Animal food |
|  |  |  | Human Food |
|  |  | Matisia cordata Bonpl. | Human Food |
|  |  |  | Sales |
|  |  | Musa paradisiaca L. | Human Food |
|  |  |  | Sales |
|  |  |  | Shade |
|  |  | Persea americana Mill. | Human Food |
|  |  |  | Shade |
|  |  | Podocarpus guatemalensis Standley | Sales |
|  |  | Pourouma cecropiifolia Mart. | Human Food |
|  |  |  | Sales |
|  |  | Saccharum officinarum L. | Human Food |
|  |  | Stryphnodendron sp | Shade |
|  |  |  | Wood |
|  |  | Zea mays L. | Human Food |
| GaCocExF | Caqueta | Acacia mangium Willd. | Ornamental |
|  |  |  | Soil conservation |
|  |  | Acrocarpus fraxinifolius Arn. | Shade |
|  |  | Alocasia macrorrhizos (L.) G. Don | Animal food |
|  |  |  | Human Food |
|  |  | Anacardium occidentale Linneo. | Shade |
|  |  | Ananas comosus L. | Human Food |
|  |  | Annona cherimola Mill. | Human Food |
|  |  | Annona muricata L. | Human Food |
|  |  | Baccharis latifolia (Ruíz & Pav.) Pers. | Shade |
|  |  | Bactris gasipaes Kunth | Human Food |
|  |  | Bertholletia excelsa Bonpl. | Wood |
|  |  | Cariniana pyriformis Miers. | Ornamental |
|  |  |  | Shade |
|  |  |  | Soil conservation |
|  |  |  | Wood |
|  |  | Cedrela odorata L. | Green Connectivity |
|  |  |  | Ornamental |
|  |  |  | Shade |
|  |  |  | Soil conservation |
|  |  |  | Wood |
|  |  | Cedrelinga catanaeformis Ducke | Ornamental |
|  |  |  | Shade |
|  |  |  | Soil conservation |
|  |  |  | Wood |
|  |  | Ceiba pentandra (L.) Gaertn. | Reforestation |
|  |  | Citrus aurantium L. | Human Food |
|  |  |  | Shade |
|  |  | Citrus limon (L.) Osbeck | Human Food |
|  |  |  | Shade |
|  |  | Clathrotropis macrocarpa Ducke | Wood |
|  |  | Coffea arabica L. | Human Food |
|  |  | Cordia alliodora (Ruíz & Pav.) Oken. | Green Connectivity |
|  |  |  | Ornamental |
|  |  |  | Shade |
|  |  |  | Soil conservation |
|  |  |  | Wood |
|  |  | Gliricidia sepium (Jacq.) Kunth ex Walp. | Medical |
|  |  |  | Shade |
|  |  | Gmelina arborea Roxb. ex Sm. | Shade |
|  |  | Guarea guidonia (L.) Sleumer. | Shade |
|  |  | Hevea brasiliensis (Willd. ex A. Juss.) Müll. Arg. | Shade |
|  |  | Iriartea deltoidea Ruiz & Pav. | Reforestation |
|  |  | Jacaranda copaia (Aubl.) D.Don. | Ornamental |
|  |  |  | Shade |
|  |  |  | Soil conservation |
|  |  |  | Wood |
|  |  | Lonchocarpus macrophyllus Kunth | Green Connectivity |
|  |  |  | Human Food |
|  |  |  | Shade |
|  |  | Mangifera indica L. | Human Food |
|  |  | Manihot esculenta Crantz | Human Food |
|  |  |  | Sales |
|  |  | Minquartia guianensis Aubl. | Shade |
|  |  | Musa paradisiaca L. | Human Food |
|  |  |  | Sales |
|  |  |  | Shade |
|  |  | Musa sapientum L. | Human Food |
|  |  | Myrcia guianensis (Aubl.) DC. | Shade |
|  |  | Nectandra acuminata (Nees & C.Mart.) J.F.Macbr. | Live fence |
|  |  | Nectandra acutifolia (Ruiz & Pav.) Mez. | Shade |
|  |  | Ochroma pyramidale (Cav. Ex Lam.) Urb. | Wood |
|  |  | Ocotea aciphylla (Nees) | Human Food |
|  |  |  | Live fence |
|  |  | Oliganthes discolor (Kunth) Sch.Bip. | Wood |
|  |  | Parkia sp | Reforestation |
|  |  | Persea americana Mill. | Human Food |
|  |  | Piptocoma discolor (Kunth) Pruski. | Shade |
|  |  |  | Wood |
|  |  | Pourouma cecropiifolia Mart. | Human Food |
|  |  | Protium guianense (Aubl.) | Reforestation |
|  |  | Psidium guajava L. | Human Food |
|  |  | Syzygium malaccense (L.) Merr. & L. M. Perry | Shade |
|  |  | Tectona grandis L.f. | Ornamental |
|  |  |  | Soil conservation |
|  |  | Trichanthera gigantea (Humb. & Bonpl.) Nees | Green Connectivity |
|  |  |  | Shade |
|  |  | Unonopsis pacifica | Wood |
|  |  | Virola flexuosa A.C. Sm. | Reforestation |
|  |  | Zygia longifolia (Willd.) Britton & Rose | Shade |
|  | Huila | Sechium edule (Jacq.) Sw. | Human Food |
|  |  | Anacardium excelsum (Bertero & Balb. ex Kunth) Skeels | Shade |
|  |  |  | Wood |
|  |  | Annona muricata L. | Human Food |
|  |  | Bambusa sp | Sales |
|  |  |  | Wood |
|  |  | Carica papaya L. | Animal food |
|  |  | Cassia grandis L.F. | Shade |
|  |  |  | Wood |
|  |  | Cedrela odorata L. | Shade |
|  |  | Citrus aurantium L. | Human Food |
|  |  | Coffea arabica L. | Sales |
|  |  | Cordia alliodora (Ruíz & Pav.) Oken. | Shade |
|  |  |  | Wood |
|  |  | Erythrina poeppigiana (Walp.) O. F. Cook | Shade |
|  |  |  | Wood |
|  |  | Gliricidia sepium (Jacq.) Kunth ex Walp. | Animal food |
|  |  |  | Live fence |
|  |  |  | Shade |
|  |  |  | Wood |
|  |  | Leucaena leucocephala (Lam.) de Wit | Wood |
|  |  | Lonchocarpus macrophyllus Kunth | Human Food |
|  |  |  | Shade |
|  |  | Maclura tinctoria (L.) D. Don ex Steud. | Shade |
|  |  |  | Wood |
|  |  | Mangifera indica L. | Human Food |
|  |  |  | Sales |
|  |  |  | Shade |
|  |  | Manihot esculenta Crantz | Human Food |
|  |  | Musa paradisiaca L. | Human Food |
|  |  |  | Sales |
|  |  | Musa sapientum L. | Animal food |
|  |  |  | Forage |
|  |  |  | Human Food |
|  |  |  | Sales |
|  |  |  | Shade |
|  |  | Myrcia guianensis (Aubl.) DC. | Shade |
|  |  |  | Wood |
|  |  | Myrsine guianensis (Aubl.) Kuntze | Shade |
|  |  |  | Wood |
|  |  | Persea americana Mill. | Human Food |
|  |  |  | Sales |
|  |  | Pseudosamanea guachapele (Kunth) Harms | Human Food |
|  |  |  | Shade |
|  |  |  | Wood |
|  |  | Tabebuia rosea (Bertol.) DC. | Shade |
|  |  | Trichanthera gigantea (Humb. & Bonpl.) Nees | Live fence |
|  |  |  | Shade |
|  | Meta | Acacia mangium Willd. | Shade |
|  |  | Annona muricata L. | Human Food |
|  |  | Cariniana pyriformis Miers. | Shade |
|  |  |  | Wood |
|  |  | Cassia grandis L.F. | Ornamental |
|  |  |  | Soil conservation |
|  |  | Cedrela odorata L. | Shade |
|  |  |  | Wood |
|  |  | Citrus aurantium L. | Human Food |
|  |  |  | Live fence |
|  |  |  | Sales |
|  |  | Citrus reticulata B. | Human Food |
|  |  |  | Sales |
|  |  | Coffea arabica L. | Sales |
|  |  | Cordia alliodora (Ruíz & Pav.) Oken. | Ornamental |
|  |  |  | Shade |
|  |  |  | Soil conservation |
|  |  | Gliricidia sepium (Jacq.) Kunth ex Walp. | Animal food |
|  |  |  | Fertilization |
|  |  |  | Medical |
|  |  |  | Shade |
|  |  | Leucaena leucocephala (Lam.) de Wit | Shade |
|  |  | Mangifera indica L. | Human Food |
|  |  |  | Shade |
|  |  | Manihot esculenta Crantz | Animal food |
|  |  | Manihot esculenta Crantz | Human Food |
|  |  | Matisia cordata Bonpl. | Human Food |
|  |  | Musa paradisiaca L. | Human Food |
|  |  |  | Shade |
|  |  | Musa sapientum L. | Human Food |
|  |  | Persea americana Mill. | Human Food |
|  |  |  | Sales |
|  |  |  | Shade |
|  |  | Pseudosamanea guachapele (Kunth) Harms | Shade |
|  |  |  | Wood |
|  |  | Saccharum officinarum L. | Human Food |
|  |  | Samanea saman (Jacq.) Merr. | Shade |
|  |  | Solanum betaceum Cav. | Human Food |
|  |  | Stryphnodendron sp | Firewood |
|  |  |  | Ornamental |
|  |  |  | Shade |
|  |  |  | Soil conservation |
|  |  | Tabebuia rosea (Bertol.) DC. | Shade |
